# 5-HT_1B_ receptor agonist enhances breakpoint for cocaine on a progressive ratio (PR) schedule during maintenance of self-administration in female rats but reduces breakpoint for sucrose

**DOI:** 10.3389/fnbeh.2022.1020146

**Published:** 2022-11-01

**Authors:** Samantha N. Scott, Brielle A. Ruscitti, Raul Garcia, Toan T. Nguyen, Kevin M. Blattner, Benjamin E. Blass, Janet L. Neisewander

**Affiliations:** ^1^School of Life Sciences, Arizona State University, Tempe, AZ, United States; ^2^School of Biological and Health Systems Engineering, Arizona State University, Tempe, AZ, United States; ^3^Moulder Center for Drug Discovery Research, Department of Pharmaceutical Sciences, Temple University School of Pharmacy, Philadelphia, PA, United States

**Keywords:** CP94253, motivation, anxiety, dependence, abstinence

## Abstract

**Background:** Previous research showed that the 5-HT_1B_ receptor agonist CP94253 enhanced cocaine reinforcement rate during maintenance of daily self-administration (SA), but inhibited reinforcement rate after 21 days of abstinence in male rats. Here we examined whether female rats show similar effects of CP94253 during maintenance as males across estrous cycle phases.

**Methods:** Female rats trained on a fixed ratio 5 (FR5) cocaine reinforcement schedule were tested for the effects of CP94253 (5.6 mg/kg, s.c.) on cocaine reinforcement rate during each phase of the estrous cycle, with access to either low (0.075 and 0.1875) or high (0.375 and 0.75) cocaine doses available for 1 h sequentially in descending dose order. Other female and male rats trained on a progressive ratio (PR) schedule of cocaine or sucrose reinforcement were tested for CP94253 (0, 3.2, 5.6, and 10 mg/kg, s.c.) effects on reinforcement rate in 3-h sessions. CP94253 effects on responding during sucrose cue-reactivity were also examined post-abstinence.

**Results:** Regardless of sex, CP94253 enhanced breakpoints on the PR schedule during maintenance of cocaine SA but attenuated breakpoints for sucrose reinforcement and decreased responding during sucrose cue-reactivity. FR results showed that CP94253 attenuated cocaine reinforcement rate during all estrous cycle phases except metestrus.

**Conclusions:** Overall, we suggest that CP94253 increased incentive motivation for cocaine during maintenance of SA in female and male rats, yet decreased motivation for sucrose. We also suggest that 5-HT_1B_Rs modulate motivation similarly across sexes except when females are in metestrus.

## Introduction

Psychostimulant use disorders are a major, persistent societal problem for which there is no approved pharmacotherapy in the United States (Pomara et al., 2012). Preclinical evidence suggests that serotonin 1B receptor (5-HT_1B_R) agonists show promise as pharmacological medications for reducing psychostimulant reinforcement. Initially this idea was counter-indicated by Parsons et al. (1998), who discovered that 5-HT_1B_R agonists shift the cocaine dose-response curve upward and to the left on a low effort fixed ratio (FR) schedule of cocaine self-administration (SA), and increase cocaine reinforcement and breakpoint on a high effort progressive ratio (PR) schedule of reinforcement in male rats, suggesting an enhancement of cocaine reinforcement and incentive motivation. However, our lab found that the effects of 5-HT_1B_R agonists on cocaine SA in males depend on whether rats have undergone a period of prolonged abstinence. Specifically, systemic administration of 5-HT_1B_R agonists or over expression of 5-HT_1B_Rs in the nucleus accumbens during maintenance of daily cocaine SA shifts the dose-response curve on an FR5 schedule of cocaine reinforcement to the left and increases breakpoint and cocaine reinforcement rate on a PR schedule in males (Pentkowski et al., 2009, 2012, Pentkowski et al., 2014). By contrast, after 21 days of forced abstinence, these manipulations shift the cocaine dose-response curve downward on a FR5 schedule, and decrease breakpoints and cocaine infusion rates on a PR schedule, suggesting a decrease in cocaine reinforcing effects and/or incentive motivation after abstinence (Pentkowski et al., 2012, 2014). Additionally, 5-HT_1B_R agonist decreases cocaine-reinforcing effects after abstinence and subsequent resumption of cocaine SA (i.e., relapse) regardless of sex (Scott et al., 2021). Moreover, 5-HT_1B_R agonists decrease both cue-elicited and cocaine-primed reinstatement of extinguished cocaine-seeking behavior in male and female rats (Acosta et al., 2005; Pentkowski et al., 2009, 2012; Scott et al., 2021). Together the findings suggest that there is an abstinence-dependent switch in 5-HT_1B_R modulation of cocaine SA in male rats from enhancement during maintenance to attenuation after a period of abstinence. However, it remains unclear whether 5-HT_1B_R agonist enhances cocaine-reinforcing effects in female rats prior to abstinence as observed previously in male rats (Pentkowski et al., 2009, 2014).

Sex differences in treatment effects are important to consider as preclinical research suggests that female rats are more sensitive to the effects of cocaine than male rats. For example, female rats acquire cocaine SA at a faster rate and consume more cocaine than male rats (Lynch and Carroll, 1999; Hu et al., 2004; Jackson et al., 2006; Lynch, 2006). Estrogens may play a crucial role in this sex difference as the exogenous administration of estradiol increases the acquisition of cocaine SA in ovariectomized (OVX) female rats when compared to OVX vehicle control females or castrated male rats (Jackson et al., 2006; Becker and Hu, 2008). During maintenance of daily cocaine SA, females obtain more infusions and reach higher breakpoints than males on a PR schedule (Carroll et al., 2002; Lynch and Taylor, 2004). Similarly, female rats demonstrate higher breakpoints during the estrus phase compared to other cycle phases, suggesting that motivation for cocaine is higher during the estrus phase (Roberts et al., 1989; Lacy et al., 2016). Additionally, female rats show greater reinstatement to a lower priming dose than males (Lynch and Carroll, 2000; Doncheck et al., 2020) with reports of heightened reinstatement of responding during cocaine cue-reactivity during proestrus (Feltenstein et al., 2011; Doncheck et al., 2018, 2021) and estrus phases (Kippin et al., 2005; Feltenstein and See, 2007). Together, these findings suggest that there are sex differences involving ovarian hormones in all phases of the cocaine addiction cycle.

This study tested the hypotheses that 5-HT_1B_R agonists: (1) enhance cocaine reinforcement rate in females tested before abstinence as observed previously in males; and (2) that sensitivity to this effect in females is particularly robust during the estrus phase of the estrous cycle when estrogens are relatively high. To test these hypotheses, we examined the effects of the 5-HT_1B_R agonist CP94253 on cocaine reinforcement rate under a FR5 schedule at each phase of the estrous cycle during maintenance of cocaine SA, and with another cohort of male and female rats, we tested CP94253 effects under a PR schedule of cocaine reinforcement. To further assess the effects of CP94253 on the ability to perform an operant response, we examined sucrose reinforcement rates under a PR schedule as well as responding during sucrose cue-reactivity tests.

## Methods

### Animals

Male and female Sprague-Dawley rats (Charles River, San Diego, CA) weighing 200–300 g at the start of the experiment were single-housed in a climate-controlled facility on a reversed light/dark cycle (10 h light/ 14 h dark cycle). Rats had *ad libitum* access to food and water except during initial cocaine SA training sessions as described below. Rats were handled for 6–7 days prior to any experimental procedure. Animal care and husbandry adhered to the Guide for the Care and Use of Laboratory Animals. All experimental procedures were approved by the Institutional Animal Care and Use Committee at Arizona State University.

### Surgery

Rats were anesthetized with isoflurane (2.5%–3.5%; VetOne, Idaho, USA) and received 0.05 mg/kg (s.c.) Buprenex (Par Pharmaceuticals, New York, USA) before surgery. A chronic indwelling catheter was then implanted into the jugular vein according to the methods previously described (Pockros et al., 2011). After surgery, rats were given cefazolin (30 mg/0.1 ml s.c.; WG Critical Care, NJ, USA), meloxicam (1 mg/kg/ml s.c.; Norbrook Laboratories Limited, Newry, Northern Ireland), and topical Neosporin ointment (NJ, USA) applied at incision sites. Rats were given 5–6 days of recovery prior to cocaine SA training. Rats received an injection of meloxicam (s.c.) the day after surgery and catheters were flushed with 0.1 ml cefazolin (10 mg/0.1 ml; i.v.) and with 0.1 ml (i.v.) saline containing heparin sodium (70 USP/1 ml; SAGENT pharmaceuticals, IL, USA) daily for 4 days during recovery. Thereafter, only heparin sodium was given daily throughout the SA experiments. Also, catheters were periodically tested for patency by administering 0.05 ml methohexital sodium (0.835 mg/0.05 ml i.v.; Jones Pharma Inc., MO, USA) at a dose that produces anesthetic effects only when administered i.v.

### Drugs

Cocaine hydrochloride was provided by the NIDA Drug Supply Program (RTI International, Research Triangle Park, NC) and was dissolved in bacteriostatic saline (Hospira Inc, Lake Forest, IL) and filtered through 0.2 μm membrane Acrodisc syringe filters (PALL Corporation, Ann Arbor, MI). CP94253 hydrochloride was purchased (Tocris Biosciences, Minneapolis, MN; Santa Cruz Biotechnology, Inc, TX, USA) or synthesized by Benjamin Blass and colleagues at Temple University. CP94253 doses are based on the salt form of drug and were prepared fresh daily using bacteriostatic saline. The volume of i.v. injections was 0.1 ml and all other injections were given at a volume of 1 ml/kg.

### Vaginal swabs

Estrous cycle phases were determined by daily vaginal cytology (Becker et al., 2005; Byers et al., 2012) using the procedures described in Peartree et al. (2017).

### Apparatus

Operant conditioning chambers (30 × 25 × 25 cm; Med Associates, St. Albans, VT) were equipped with a ventilation fan, an active and inactive lever, a cue light above the active lever, a house light, and a tone generator as previously described (Pentkowski et al., 2009). Infusion pumps were located outside of a sound-attenuating outer chamber. Polyethylene tubing attached to the drug syringe in the infusion pump entered through a port in the outer chamber wall and attached to a liquid swivel (Instech, Plymouth, PA) suspended above the inner chamber. The outlet line from the liquid swivel was encased inside a metal spring leash with a plastic connector that fastened to the catheter (Plastics One, Roanoke, VA).

### Experiment 1: effects of the estrous cycle and CP94253 on maintenance of cocaine SA on an FR5 schedule of reinforcement

Forty-eight hours prior to the first SA session, rats were food-restricted to 85% of their initial free-feeding body weight to facilitate the acquisition of cocaine SA. Two-hour SA sessions occurred at approximately the same time of day 6 days/week during the rats’ dark cycle. Cocaine (0.75 mg/kg/0.1 ml infusion, i.v.) was available initially on a fixed ratio (FR) 1 schedule of reinforcement that progressed to an FR2, FR3, and FR5 based on individual performance. Ratio advancement within a session occurred once rats received seven cocaine reinforcers within an hour. The successful completion of a reinforcement schedule activated the tone and light cues and 1 s later activated the infusion pump to deliver cocaine over 6 s. These stimuli were then terminated and a house light was illuminated for 20 s to signal a timeout during which lever responses were recorded but produced no consequences. Once rats progressed to the FR5, they were given *ad libitum* access to food and began receiving daily vaginal swabs 30 min after the sessions to track their estrous cycle phases. Testing began once rats displayed stable cocaine reinforcement rate, defined as ≤15% variability in the number of infusions obtained across three consecutive sessions.

The rats were tested twice at each phase of the estrous cycle, receiving CP94253 (5.6 mg/kg, s.c.) prior to one test and vehicle prior to the other test, with the order of pretreatment counterbalanced. Fifteen minutes after the treatment injection, rats were placed into the SA chambers with cocaine available on an FR5 schedule of reinforcement. Separate groups of rats were tested on different dates but at the same time of day either with 0.075 and 0.1875 mg/kg (cohort 1, *n* = 19) or with 0.375 and 0.75 mg/kg (cohort 2, *n* = 35) cocaine available for 1-h each in descending order of dose with a 5-min time out between doses. Rats were given at least three additional SA sessions between tests to re-establish the stability criterion. Thirty min after each test, predicted estrous cycle phase was verified.

### Experiment 2: effects of CP94253 on maintenance of cocaine SA on a PR schedule of reinforcement in males and females

A new cohort of male (*n* = 9) and female (*n* = 16) rats were food-restricted and trained to self-administer 0.75 mg/kg, i.v. cocaine on a FR1 schedule of reinforcement in 3-h SA sessions 6 days/week. After establishing stable cocaine reinforcement, rats continued daily SA training during 3-h sessions with 0.375 mg/kg, i.v. cocaine available on the PR schedule (Richardson and Roberts, 1996). The reason for lowering the cocaine dose was because most rats reach breakpoint prior to the 3-h session limit at this dose. Break point is defined as the last successful schedule of reinforcement completed prior to a 1-h period during which a rat fails to complete the next schedule. Testing commenced after rats met the stability criterion on the PR schedule. Test sessions were identical to training sessions except that rats received s.c. administration of their assigned drug treatment 15 min before the session. First, rats were tested with vehicle or 5.6 mg/kg, s.c., CP94253 on separate test days with order counterbalanced. After testing at both of these doses, rats underwent two more tests after receiving 3.2 or 10 mg/kg, s.c. CP94253, counterbalanced for dose order. Rats received additional sessions between the tests to re-establish the stability criterion. This dose order was selected to maximize the number of rats tested at the 5.6 mg/kg dose (i.e., the lowest effective dose in male rats) because of concerns for attrition due to loss of catheter patency. All female rats were vaginally swabbed 30 min after each test session.

### Experiment 3: effects of CP94253 on sucrose reinforcement under a PR schedule and on sucrose cue reactivity

Once male (*n* = 8) and female (*n* = 15) rats in Experiment 2 completed testing for the effects of CP94253 on a PR schedule of cocaine reinforcement, they were placed in a different operant chamber with sucrose (45 mg pellets; Sigma Aldrich, MO, USA) available on the PR schedule. These new chambers were located in a different room and the positions of the active and inactive levers were opposite to that during cocaine SA (e.g., if the active lever had been on the right side, it was switched to the lever on the left side). Three-hour training sessions occurred 6 days/week until the stability criterion was met. Testing then commenced with rats receiving vehicle, 3.2, 5.6, or 10 mg/kg, s.c., CP94253 across tests using the identical order described for Experiment 2. After the last test, rats remained in their home cage for 10 days and then underwent testing for sucrose cue reactivity. Rats were tested twice for cue reactivity, receiving an injection of vehicle 15 min prior to one test and CP94253 (10 mg/kg, s.c.) 15 min prior to the other test, counterbalanced for order. During these 1-h test sessions, the light and tone cues previously presented with sucrose reinforcement were available on an FR1 schedule but no sucrose pellets were available. Rats remained in their home cages for 5 days intervening in the two tests.

### Statistical analyses

Statistical analyses were conducted with IBM SPSS Statistics 23.0. Infusions, active and inactive lever responses were analyzed in separate ANOVAs with cocaine dose and drug pretreatment as within subjects factors where appropriate. Although we had designed Experiment 1 with an estrous cycle phase as a repeated measure, we included this variable as a between subjects factor because there was too much missing data due to the catheter failure of rats during the testing phase. The data from rats that had been tested multiple times under the same condition due to mistaken predictions of the estrous cycle phase were averaged and the average value was used in the ANOVA. Bonferroni *post-hoc* tests were used to identify the source of significant effects.

## Results

### Baseline cocaine SA measures across estrous cycle phases

**Figure 1:**
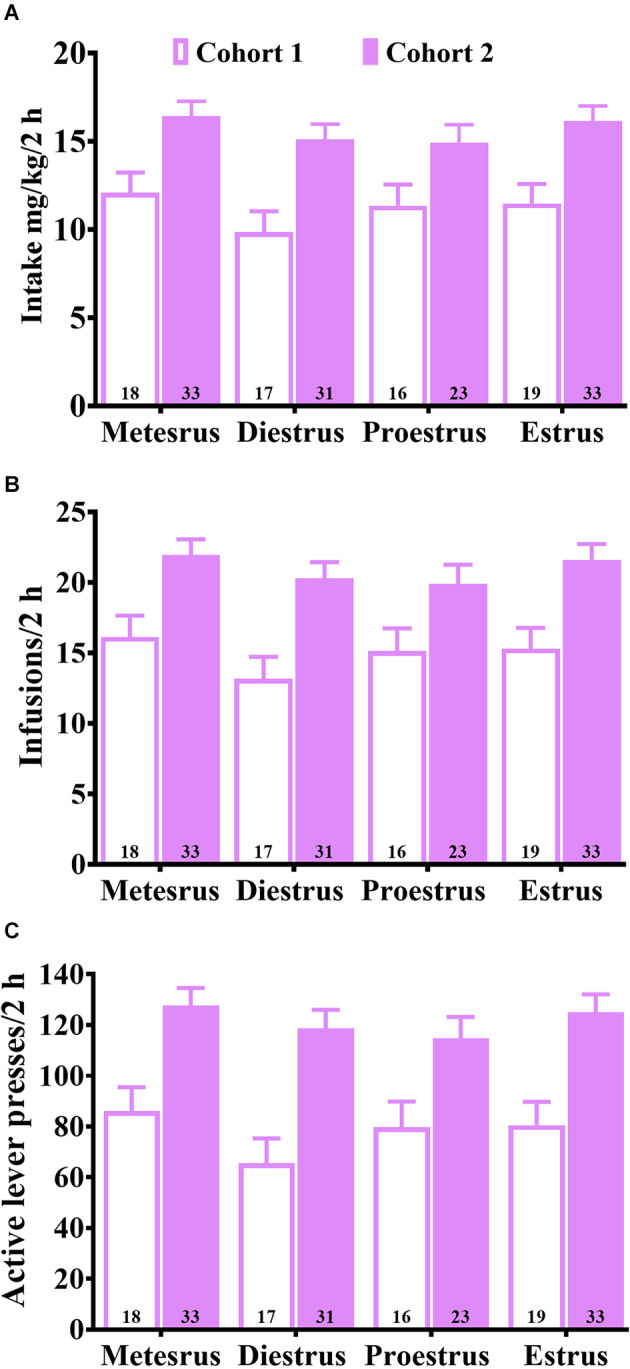
Average baseline (mean ± SEM) of daily cocaine intake (mg/kg) **(A)**, infusion rates **(B)**, and active lever response rates **(C)** during training for cohorts 1 and 2 at each estrous cycle phase measured prior to the CP94253 test phase. Both cohorts were given access to 0.75 mg/kg, i.v. cocaine on an FR5 schedule of reinforcement during training. The estrous cycle phase did not affect any of the measures.

Figure 1 shows cohort differences in baseline maintenance of daily cocaine SA on the FR5 schedule of reinforcement prior to the first CP94253 test regardless of the estrous cycle phase. The ANOVAs showed a significant main effect of dose cohort for mg/kg intake, the number of infusions, and active lever responses (*F*_(1,182)_ = 37.5, 38.1, 51.2, respectively, *p* < 0.05) due to cohort 1 showing higher measures compared to cohort 2. The reason for these differences is unclear but is likely due to running the cohorts during different months. There was no main effect of the estrous cycle phase nor interaction with dose cohort for any of the measures (Figures 1A–C), including inactive lever responses (data not shown).

### Effects of estrous cycle and CP94253 on maintenance of cocaine SA on the FR5 schedule of reinforcement

**Figure 2:**
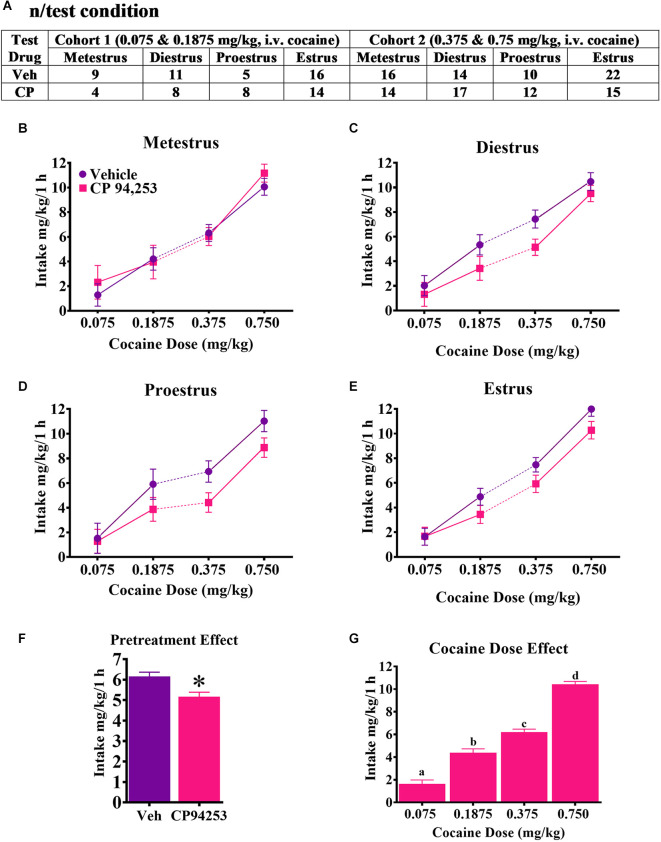
The number of rats tested in each condition under an FR5 reinforcement schedule **(A)** and effects of CP94253 on their mg/kg cocaine intake (mean ± SEM) during metestrus **(B)**, diestrus **(C)**, proestrus **(D)** and estrus **(E)**. Cohort 1 had access to 0.075 and 0.1875 mg/kg, i.v. cocaine in descending order during the test phase of the experiment whereas cohort 2 had access to 0.375 and 0.75 mg/kg, i.v. cocaine in descending order. For subjects tested more than once under the same condition due to mis-predicted estrous cycle phase, the average of the tests was used in the ANOVA. There were no effects nor interactions with the estrous cycle phase. A main effect of pretreatment **(F)** indicated less mg/kg cocaine intake after CP94253 pretreatment compared to the vehicle regardless of cocaine dose and a main effect of dose indicated that rats consumed more cocaine as the dose available increased **(G)**. *Indicates a significant difference from the vehicle, ANOVA main effect, *p* < 0.05. “a”, “b”, “c”, and “d” letter symbols indicate a significant difference from each other, Bonferroni *t*-tests, *p* < 0.0167.

CP94253 effects on mg/kg cocaine intake during the maintenance of daily SA in each phase of the estrous cycle of female rats is shown in Figure 2. ANOVA of cocaine intake showed no effect of estrous phase nor interaction with pretreatment or cocaine dose (Figures 2B–E). There were main effects of pretreatment (*F*_(1,358)_ = 10.6, *p* < 0.05) and cocaine dose (*F*_(3,358)_ = 156.4, *p* < 0.05). Rats consumed less cocaine after receiving CP94253 pretreatment compared to the vehicle regardless of cocaine dose and they consumed more cocaine as the dose increased (Figures 2F,G, respectively; Bonferroni *t*-tests, *p* < 0.0167).

**Figure 3:**
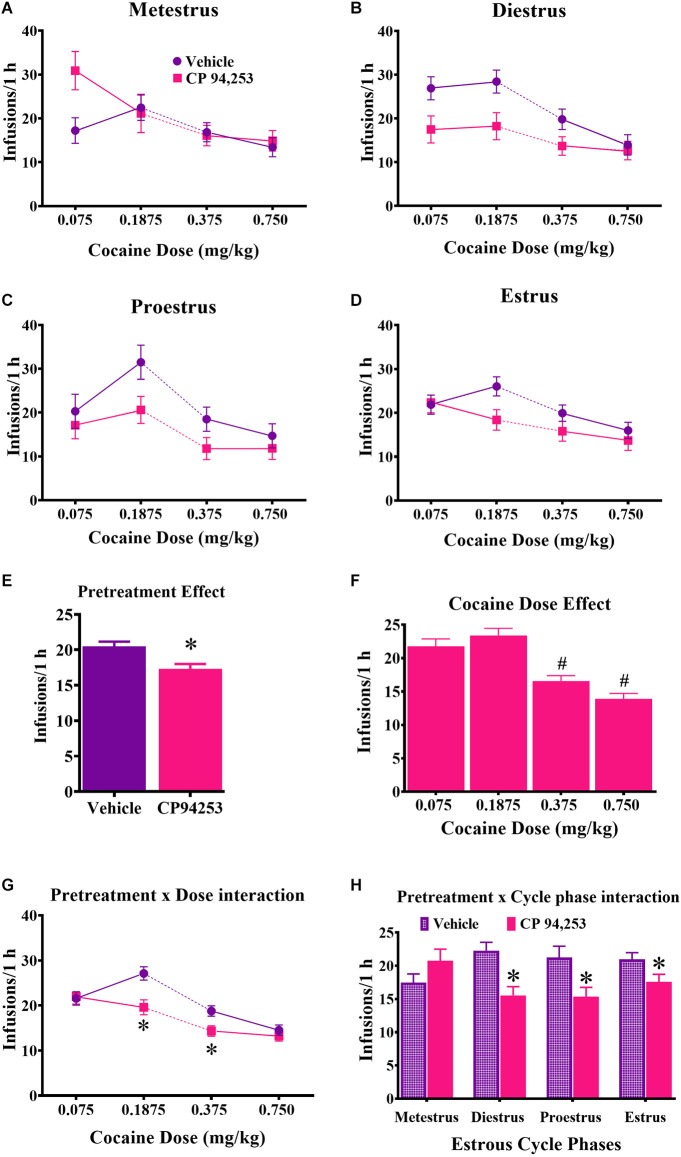
Effects of the 5-HT_1B_R agonist CP94253 on cocaine infusion rate (mean ± SEM) under an FR5 reinforcement schedule in female rats during metestrus **(A)**, diestrus **(B)**, proestrus **(C)**, and estrus **(D)**, and ANOVA main effects of pretreatment **(E)** and dose **(F)** and interactions between pretreatment and dose **(G)** and pretreatment and cycle phase **(H)**. For experimental details and n/condition see text or Figure 1 caption. *Difference from vehicle pretreatment, ANOVA main effect, *p* < 0.05 shown collapsed across pretreatment and estrous cycle phase or *post-hoc* Bonferroni *t*-tests, *p* < 0.0167; ^#^difference from 0.075 and 0.1875 cocaine doses, Bonferroni *t*-tests, *p* < 0.0167.

Reinforcement rates under the FR5 schedule of reinforcement are shown in Figure 3. ANOVA of infusions obtained showed the main effects of pretreatment (*F*_(1,358)_ = 10.8, *p* < 0.05) and cocaine dose (*F*_(3,358)_ = 21.0, *p* < 0.05), but no main effect of estrous cycle. Rats pretreated with CP94253 took fewer cocaine infusions compared to vehicle-pretreated rats (Figure 3E). The dose effect was due to fewer infusions earned at the two higher doses compared to the two lower doses (Figure 3F; Bonferroni *t*-tests, *p* < 0.0167). Interestingly, there were pretreatment by cocaine dose and pretreatment by estrous cycle phase interactions (*F*_(3,358)_ = 2.8, 4.7, respectively, *p* < 0.05), but no 3-way interaction. *Post-hoc* analyses indicated that CP94253 pretreatment decreased infusions at intermediate cocaine doses (0.1875 and 0.375 mg/kg, i.v.) compared to vehicle pretreatment (Figure 3G; Bonferroni *t*-test, *p* < 0.0063) and when collapsed across all cocaine doses, CP94253 reduced the number of infusions obtained at all estrous cycle phases except metestrus (Figure 3H; *p* < 0.0063). The latter suggests that CP94253 was less effective during metestrus.

**Figure 4:**
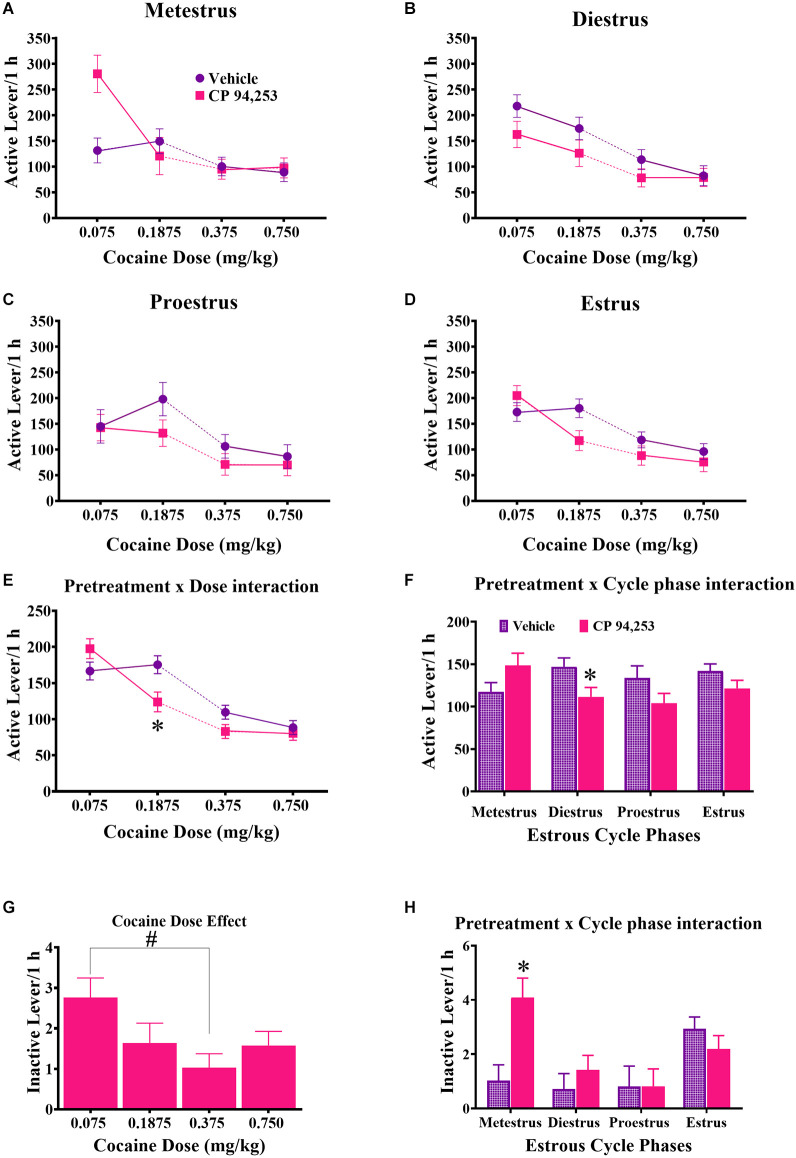
Effects of CP94253 on mean ± SEM active lever response rates **(A–F)** and inactive lever response rates **(G,H)** under an FR5 reinforcement schedule in female rats during each estrous cycle phase **(A–D)** and ANOVA interactions between pretreatment and dose **(E)** and pretreatment and cycle phase **(F)** for active lever responses and the main effect of dose **(G)** and pretreatment by cycle phase interaction **(H)** for inactive lever responses. For experimental details and n/condition see text or Figure 1 caption. *Difference from vehicle pretreatment, Bonferroni *t*-tests, *p* < 0.0167; ^#^difference between 0.075 and 0.375 mg/kg cocaine, Bonferroni *t*-tests, *p* < 0.0167.

ANOVA of active lever responses showed no three-way interaction (Figures 4A–D), but as expected there was a main effect of cocaine dose (*F*_(3,358)_ = 31.5, *p* < 0.05) and *post-hoc* Bonferroni *t*-tests (*p* < 0.0125) showed that active lever response rates were lower at the two high doses compared to the two low doses. There were also pretreatment by cocaine dose and pretreatment by estrous cycle phase interactions (*F*_(3,358)_ = 3.7, 3.0, respectively, *p* < 0.05). Subsequent *post-hoc* analyses showed that pretreatment with CP94253 decreased response rates at 0.1875 mg/kg, i.v. cocaine dose compared to vehicle pretreatment (Figure 4E; Bonferroni *t*-test, *p* < 0.0063). CP94253 also decreased active lever response rates during diestrus compared to vehicle, but there were no effects at other estrous cycle phases (Figure 4F, Bonferroni *t*-test, *p* < 0.0063). ANOVA of inactive lever responses revealed a main effect of cocaine dose, estrous phase, and a pretreatment by estrous phase interaction (*F*_(3,317)_ = 2.81, 5.18, 3.85, respectively, *p* < 0.05). The rats displayed higher inactive lever responses when 0.075 mg/kg, i.v. was available compared to 0.375 mg/kg, i.v. cocaine (Figure 4G, Bonferroni *t*-test, *p* < 0.0125), and CP94253 pretreatment increased inactive lever responses only during metestrus (Figure 4H, Bonferroni *t*-test, *p* < 0.0063).

### Effects of CP94253 on maintenance of cocaine SA on a PR schedule of reinforcement in males and females

**Figure 5:**
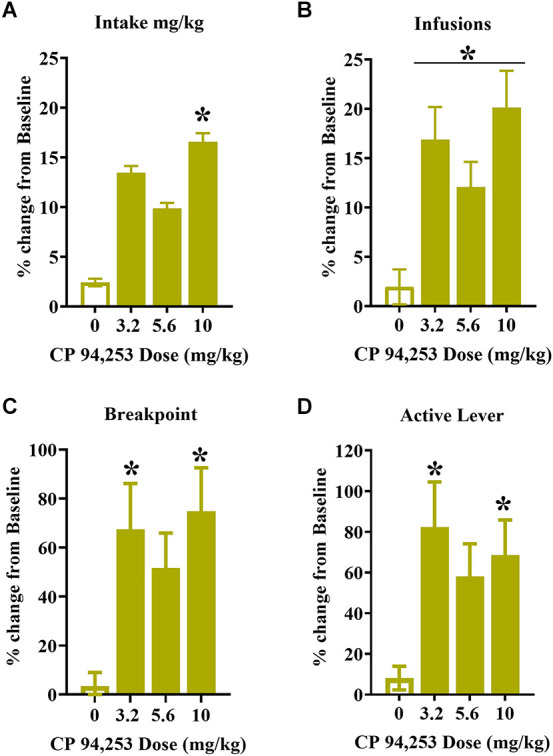
Effects of CP94253 on the mean ± SEM mg/kg cocaine intake **(A)**, cocaine infusions **(B)**, breakpoints **(C)**, and active lever responses **(D)** during maintenance of daily cocaine self-administration (SA) under a progressive ratio (PR) schedule of reinforcement expressed as a % change from training baseline in male (*n* = 9) and female (*n* = 16) rats. There were no main effects or interactions with sex, therefore the data are collapsed across sex. *Difference from vehicle, Bonferroni *t*-tests, *p* < 0.0167.

To control for variability in testing measures, the results were analyzed as a percent change from individual baselines. There were no effects of sex on any of the measures; therefore, these data are shown collapsed across male and female rats (Figures 5A–C). The ANOVA of mg/kg cocaine intake showed a main effect of pretreatment (*F*_(2.6,55)_ = 5.19, *p* < 0.001), where 10 mg/kg CP94253 enhanced cocaine intake (mg/kg) compared to all other conditions (Bonferroni *t*-tests, *p* < 0.0125, Figure 5A). ANOVA of infusions obtained showed a similar main effect of pretreatment (*F*_(2.4,56)_ = 8.37, *p* < 0.001), where all doses of CP94253 (3.2, 5.6, and 10 mg/kg) enhanced cocaine infusions obtained when compared to vehicle (Bonferroni *t*-tests, *p* < 0.0125, Figure 5B). Lastly, there were main effects of pretreatment for breakpoint (*F*_(2.6,68)_ = 5.95, *p* < 0.05, Figure 5C) and active lever responses (*F*_(2.19,55)_ = 4.66, *p* < 0.05, Figure 5D), which were due to increases in these measures after administration of 3.2 and 10 mg/kg CP94253 (Bonferroni *t*-tests, *p* < 0.0125). There were no significant effects nor interactions for inactive lever responses (data not shown).

### Effects of CP94253 on sucrose reinforcement under a PR schedule of reinforcement in males and females

**Figure 6:**
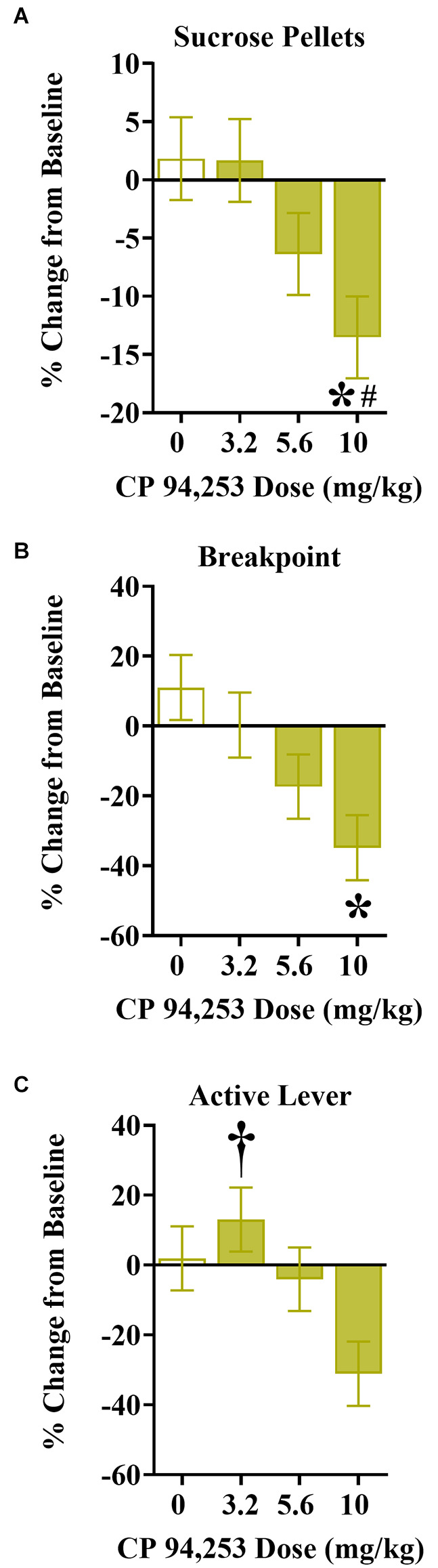
Effects of CP94253 on mean ± SEM sucrose reinforcers earned **(A)**, breakpoints **(B)**, and active lever responses **(C)** under a PR schedule of reinforcement expressed as a % change from training baseline in male (*n* = 8) and female (*n* = 15) rats. There were no main effects or interactions with sex, therefore the data are collapsed across sex. *Difference from vehicle, Bonferroni *t*-tests, *p* < 0.0167; ^#^difference from 3.2 mg/kg CP94253, Bonferroni *t*-tests, *p* < 0.0167; ^†^different from 10 mg/kg CP94253, Bonferroni *t*-tests, *p* < 0.0167.

The effects of CP94253 on sucrose reinforcement measures are shown in Figure 6. There were no effects of sex, therefore the data are collapsed across sex (Figures 6A–C). ANOVA of sucrose pellets earned showed a main effect of pretreatment (*F*_(3,81)_ = 3.39, *p* < 0.05), due to a decrease in the number of sucrose reinforcers obtained at the highest dose of CP94253 (10 mg/kg) when compared to all other CP94253 doses (Bonferroni *t*-tests, *p* < 0.0125, Figure 6A). ANOVA of breakpoints showed a similar main effect of pretreatment (*F*_(3,80)_ = 4.41, *p* < 0.05), where 10 mg/kg CP94253 decreased the breakpoint for sucrose reinforcers in comparison to vehicle (Bonferroni *t*-tests, *p* < 0.0125, Figure 6B). Lastly, ANOVA of active lever responses for sucrose showed a main effect of pretreatment (*F*_(3,81)_ = 3.61, *p* < 0.05), revealing that 3.2 mg/kg CP94253 increased active lever responses for sucrose compared to 10 mg/kg CP94253 (Bonferroni *t*-tests, *p* < 0.0125, Figure 6C). There were no significant effects on inactive lever responses for sucrose reinforcement (data not shown).

### Effects of CP94253 on responding during sucrose cue-reactivity tests

**Figure 7:**
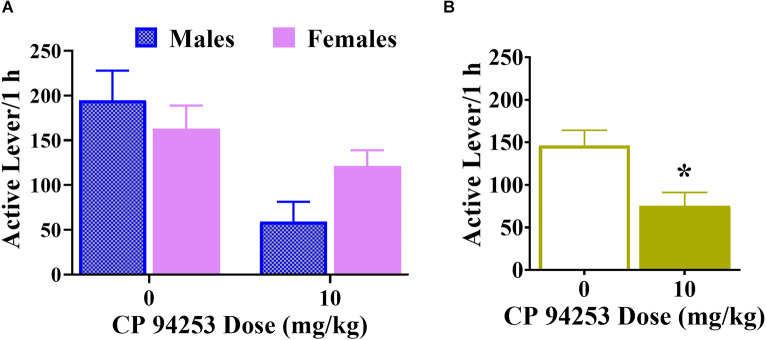
Effects of CP94253 (10 mg/kg, SC) on responding during sucrose cue-reactivity test expressed as the mean ± SEM active lever presses in male (*n* = 8) and female (*n* = 15) rats **(A)** and in all rats **(B)**. Testing took place at least 10 days after the last training session. There were no main effects or interactions with sex. *Difference from vehicle, Bonferroni *t*-tests, *p* < 0.0167.

ANOVA of effects of CP94253 on sucrose cue reactivity assessed in males and females 10 days after the last training session showed no effect of sex (Figure 7A) but revealed a main effect of pretreatment (*F*_(3,81)_ = 3.61, *p* < 0.05) that was due to decreased active lever responses with CP94253 pretreatment compared to the vehicle regardless of sex (Bonferroni *t*-tests, *p* < 0.0125, Figure 7B). There were no significant effects on inactive lever responses (data not shown).

## Discussion

The present study examined the effects of a 5-HT_1B_ receptor agonist, CP94253, on cocaine abuse-like behavior during the maintenance of cocaine SA in rats, and whether these effects are influenced by the estrous cycle phases in female rats. The finding that cocaine reinforcement rate did not differ across phases of the estrous cycle (Figure 1) is discrepant with previous studies demonstrating greater cocaine reinforcement rate during estrus when estrogen levels are relatively high compared to diestrus and metestrus phases (Roberts et al., 1989; Hecht et al., 1999; Lynch et al., 2000; Hu et al., 2004; Feltenstein and See, 2007). The reason for this discrepancy is unclear but may be related to the use of a low-effort, FR5 schedule of reinforcement. Previous research suggests that estrous cycle effects are more reliably observed under PR compared to FR schedules, suggesting that low-effort FR schedules may lack the sensitivity to detect estrous cycle-dependent effects on cocaine SA (Roberts et al., 1989; Lynch and Taylor, 2004, 2005; Lynch, 2008; Lacy et al., 2016). Additionally, our study was longer than most cocaine SA experiments, which may have obscured estrous cycle effects because chronic cocaine SA results in irregular estrous cycles (Roberts et al., 1989; King et al., 1990; Feltenstein and See, 2007). Lastly, the discrepancy may be attributed to the dose of intravenous cocaine as estrous cycle effects are more reliably observed in studies that examine lower doses of cocaine than those that examine higher doses. For instance, female rats display higher reinforcement rates during estrus than other phases when they self-administer low doses (0.2–0.5 mg/kg, i.v.) of cocaine (Roberts et al., 1989; Hecht et al., 1999; Lynch et al., 2000; Hu et al., 2004; Feltenstein and See, 2007), but not high doses such as 0.75–1.5 mg/kg, i.v. cocaine (present study; Lynch and Taylor, 2004).

Importantly, the metestrus phase of the estrous cycle impacted the effects of CP94253 on cocaine SA measures. Specifically, CP94253 decreased infusion rates during all phases of the estrous cycle except metestrus (Figure 3H) and although the interaction of CP94253 and estrous cycle phase for mg/kg cocaine intake did not reach significance, it appears that the main effect of CP94253 on this measure was carried by small decreases observed at all phases except metestrus (Figures 2B–F). Furthermore, CP94253 increased inactive lever presses (Figure 4H) and produced a nonsignificant increase in active lever presses only during metestrus, whereas CP94253 decreased active lever presses during all other phases although this latter effect was only significant during diestrus. The pattern of intake, reinforcement rates, and active lever presses across estrous cycle phases overall suggests that reinforcement-related behaviors in female rats are differentially affected by CP94253 during metestrus compared to other phases of the estrous cycle. The behavior observed during metestrus may involve a nonspecific increase in activity given that inactive lever presses were increased by CP94253 during testing on the FR5 schedule of cocaine reinforcement. In any case, this estrous cycle effect is likely mediated by hormonal changes. Cocaine alters anterior pituitary hormones, such as follicle stimulating hormone (FSH) and luteinizing hormone (LH), which in turn regulate gonadal hormones across species, including humans (Mello et al., 1990, 1993; Dada and Horacek, 1991; Heesch et al., 1996; Mendelson et al., 2001). Therefore, it is possible that CP94253 alters cocaine-induced changes in FSH and LH as the ratio of these and other ovarian hormones are markedly different during metestrus compared to any other cycle phase. Further research is needed to verify and extend the present estrous cycle findings due to the low number of animals tested (e.g., *n* = 4) at some of the phases and the tendency for most initial tests to occur during estrous or metestrus, which may be problematic if the sensitivity to effects of CP94253 change with repeated testing.

The present results from the FR5 experiment showing that pretreatment with CP94253 attenuated cocaine reinforcement rates during maintenance of SA in female rats are difficult to interpret due to the inverted U-shape of the cocaine SA dose-response curve observed with low ratio schedules, and this study failed to capture the ascending limb of this curve. An increase in cocaine infusions at doses on the ascending limb of the curve with a decrease in infusions at doses on the descending limb represents a leftward shift that is typically interpreted as an enhancement or sensitization of the reinforcing effects of cocaine (Mello and Negus, 1996; Parsons et al., 1998; Piazza et al., 2000) Previous research comparing vehicle and CP94253 pretreatments showed that the highest reinforcement rate in males was observed under CP94253 pretreatment at the lowest dose of cocaine tested, which is on the ascending limb (*upward shift*), but CP94253 also decreased reinforcement rate across doses on the descending limb of the dose-response curve (*leftward shift*). This pattern was interpreted as a CP94253-induced enhancement of the reinforcing value of cocaine (Parsons et al., 1998; Pentkowski et al., 2009). In the present study, however, CP94253 effects in females resemble the males at doses considered to be on the descending limb of the dose-response curve but do not resemble males at the lowest dose tested, which is considered to be on the ascending limb. Since females failed to show an increase in reinforcement rate at the lowest dose tested, it is ambiguous whether the CP94253-induced decreases in SA measures on the descending limb were due to a decrease in cocaine reinforcing value resulting in less motivation for cocaine or to the enhancement of the reinforcing value of cocaine producing longer interdose interval due to a transient satiation effect (Killeen and Reilly, 2001; Lynch and Carroll, 2001).

To further investigate whether CP94253 enhances reinforcement/motivation for cocaine during maintenance of daily SA in female rats as observed previously in males, we measured its effects under a PR schedule of reinforcement. The PR schedule typically produces a linear increase in infusions earned across doses that are self-administered (Hodos, 1961; Depoortere et al., 1993), and therefore, increases in the number of infusions earned suggest enhancement of cocaine reinforcement/motivation, whereas decreases suggest attenuation of these processes. The results showed that CP94253 enhanced mg/kg cocaine intake, infusions, breakpoint, and active lever responses (Figure 5), supporting the hypothesis that pretreatment with CP94253 enhances the reinforcing value of cocaine in female rats during the maintenance of SA. One unusual aspect of the CP94253 dose-response curve under the PR schedule of reinforcement is that the 5.6 mg/kg dose failed to produce a significant increase in mg/kg intake, breakpoint, or active lever presses, although there were trends toward these effects as well as a significant increase in the number of infusions obtained. The inconsistent CP94253 dose-response functions may be due to a test order effect given that 5.6 mg/kg CP94253 and vehicle were always tested across the first two tests in counterbalanced order, while 3.2 and 10 mg/kg CP94253 were tested subsequently in counterbalanced order. It is possible that rats became more sensitive to CP94253 effects across repeated tests resulting in more reliable effects at the last two doses tested.

Together with previous literature, the present findings suggest that male and female rats exhibit similar abstinence-dependent CP94253 effects on cocaine reinforcement. In light of the enhancement of cocaine reinforcement/motivation under the PR schedule in both male and female rats, the most parsimonious interpretation of the CP94253-induced decrease in SA measures across increasing doses of cocaine under the FR5 schedule is that CP94253 enhanced cocaine reinforcing value/motivation in female rats, similar to the effects observed previously in males tested with 5-HT_1B_R agonist during maintenance of cocaine SA under an FR5 schedule (Parsons et al., 1998; Pentkowski et al., 2014). Reinforcement rates at 0.075 mg/kg, i.v. cocaine under an FR5 schedule are enhanced in male rats during maintenance of cocaine SA but attenuated after prolonged forced abstinence (Pentkowski et al., 2014). Recently, we replicated the latter effect in both male and female rats and showed that the effect persists even when rats are tested after resuming sessions of daily access following abstinence (Scott et al., 2021). These same rats also showed a decrease in cocaine cue-reactivity in response to CP94253 when tested after either 21 or 60 days of abstinence (Scott et al., 2021). Collectively, the findings suggest that the functional effects of 5-HT_1B_R agonists on cocaine reinforcement/motivation flip from facilitatory during maintenance of daily SA to inhibitory after prolonged abstinence in both males and females.

The effects of CP94253 on sucrose reinforcement were examined to assess possible agonist effects on the ability to perform an operant response or on consummatory behaviors in general. During maintenance of daily sessions, the highest dose of CP94253 (10 mg/kg) produced opposite effects on reinforcement rates under a PR schedule of cocaine reinforcement (i.e., increase; Figure 5) vs. sucrose reinforcement (i.e., decrease; Figure 6) regardless of sex. CP94253 (10 mg/kg) also decreased responding in both sexes during a sucrose cue-reactivity test that occurred after 10 days of abstinence (Figure 7). These findings suggest that CP94253 decreases motivation for sucrose regardless of abstinence or sex. Previous research has shown that 5-HT_1B_R agonists either decrease sucrose reinforcement and responding during sucrose cue-reactivity (Lee and Simansky, 1997; Acosta et al., 2005), or failed to alter sucrose consumption (Pentkowski et al., 2009; Garcia et al., 2020). Overall, these findings suggest that 5-HT_1B_R agonists may facilitate satiety associated with sucrose reinforcers or may decrease motivation to seek sucrose reinforcement.

In conclusion, the present results suggest that female rats show a similar enhancement of cocaine reinforcement/motivation as males in response to CP94253 given during maintenance of cocaine SA, whereas both sexes show a decrease in sucrose reinforcement/motivation in response to CP94253. The ability of CP94253 to modulate cocaine reinforcement may depend on estrous cycle phase as rats in metestrus showed different dose-response functions for cocaine SA measures compared to other phases of the estrous cycle. Because previous findings suggest that 5-HT_1B_ agonists persistently attenuate motivation for cocaine after prolonged abstinence regardless of sex (Acosta et al., 2005; Pentkowski et al., 2009, 2014; Garcia et al., 2020; Scott et al., 2021), further research is needed to understand the mechanism underlying the switch in 5-HT_1B_R agonist effects on cocaine SA during abstinence because their post-abstinence inhibition of cocaine SA suggests potential therapeutic effects for treating psychostimulant use disorders (Callahan and Cunningham, 1995; Fletcher and Korth, 1999; Miszkiel et al., 2011; Pentkowski et al., 2014; Garcia et al., 2017).

## Data Availability Statement

The original contributions presented in the study are included in the article. Further inquiries can be directed to the corresponding author.

## Ethics Statement

The animal study was reviewed and approved by Arizona State University Institutional Animal Care and Use Committee.

## Author Contributions

SS and JN designed the studies and wrote the manuscript. SS, BR, RG, and TN performed experiments. KB and BB synthesized the 5-HT_1B_R agonist used in experiments. SS analyzed the data. All authors contributed to the article and approved the submitted version.

## Funding

This study was funded by the National Institutes on Drug Abuse (DA011064, DA055153).

## References

[B1] Acosta J. I. Boynton F. A. Kirschner K. F. Neisewander J. L. (2005). Stimulation of 5-HT_1B_ receptors decreases cocaine- and sucrose-seeking behavior. Pharmacol. Biochem. Behav. 80, 297–307. 10.1016/j.pbb.2004.12.001 15680183

[B3] Becker J. B. Arnold A. P. Berkley K. J. Blaustein J. D. Eckel L. A. Hampson E.. (2005). Strategies and methods for research on sex differences in brain and behavior. Endocrinology 146, 1650–1673. 10.1210/en.2004-1142 15618360

[B2] Becker J. B. Hu M. (2008). Sex differences in drug abuse. Front. Neuroendocrinol. 29, 36–47. 10.1016/j.yfrne.2007.07.003 17904621 PMC2235192

[B4] Byers S. L. Wiles M. V. Dunn S. L. Taft R. A.. (2012). Mouse estrous cycle identification tool and images. PLoS One 7:e35538. 10.1371/journal.pone.0035538 22514749 PMC3325956

[B5] Callahan P. M. Cunningham K. A. (1995). Modulation of the discriminative stimulus properties of cocaine by 5-HT_1B_ and 5-HT_2C_ receptors. J. Pharmacol. Exp. Ther. 274, 1414–1424. 7562516

[B6] Carroll M. E. Morgan A. D. Lynch W. J. Campbell U. C. Dess N. K. (2002). Intravenous cocaine and heroin self-administration in rats selectively bred for differential saccharin intake: phenotype and sex differences. Psychopharmacology (Berl) 161, 304–313. 10.1007/s00213-002-1030-5 12021834

[B313] Dada M. O. Horacek M. J. (1991). Acute effects of intravenous administration of cocaine on circulating luteinizing hormone, follicle-stimulating hormone and prolactin concentrations in rats. Res. Commun. Subst. Abuse 12, 197–208.

[B7] Depoortere R. Y. Li D. H. Lane J. D. Emmett-Oglesby M. W. (1993). Parameters of self-administration of cocaine in rats under a progressive-ratio schedule. Pharmacol. Biochem. Behav. 45, 539–548. 10.1016/0091-3057(93)90503-l 8332614

[B8] Doncheck E. M. Anderson E. M. Konrath C. D. Liddiard G. T. DeBaker M. C. Urbanik L. A.. (2021). Estradiol regulation of the prelimbic cortex and the reinstatement of cocaine seeking in female rats. J. Neurosci. 41, 5303–5314. 10.1523/JNEUROSCI.3086-20.2021 33879537 PMC8211550

[B9] Doncheck E. M. Liddiard G. T. Konrath C. D. Liu X. Yu L. Urbanik L. A.. (2020). Sex, stress and prefrontal cortex: influence of biological sex on stress-promoted cocaine seeking. Neuropsychopharmacology 45, 1974–1985. 10.1038/s41386-020-0674-3 32303052 PMC7547655

[B10] Doncheck E. M. Urbanik L. A. Debaker M. C. Barron L. M. Liddiard G. T. Tuscher J. J.. (2018). 17β-estradiol potentiates the reinstatement of cocaine seeking in female rats: role of the prelimbic prefrontal cortex and cannabinoid type-1 receptors. Neuropsychopharmacology 43, 781–790. 10.1038/npp.2017.170 28825421 PMC5809785

[B12] Feltenstein M. W. Henderson A. R. See R. E. (2011). Enhancement of cue-induced reinstatement of cocaine-seeking in rats by yohimbine: sex differences and the role of the estrous cycle. Psychopharmacology (Berl) 216, 53–62. 10.1007/s00213-011-2187-6 21308466 PMC3195378

[B13] Fletcher P. J. Korth K. M. (1999). Activation of 5-HT_1B_ receptors in the nucleus accumbens reduces amphetamine-induced enhancement of responding for conditioned reward. Psychopharmacology (Berl) 142, 165–174. 10.1007/s002130050876 10102769

[B11] Feltenstein M. W. See R. E. (2007). Plasma progesterone levels and cocaine-seeking in freely cycling female rats across the estrous cycle. Drug Alcohol Depend. 89, 183–189. 10.1016/j.drugalcdep.2006.12.017 17240083 PMC2099261

[B14] Garcia R. Cotter A. R. Leslie K. Olive M. F. Neisewander J. L. (2017). Preclinical evidence that 5-HT_1B_ receptor agonists show promise as medications for psychostimulant use disorders. Int. J. Neuropsychopharmacol. 20, 644–653. 10.1093/ijnp/pyx025 28444326 PMC5570061

[B15] Garcia R. Le T. Scott S. N. Charmchi D. Sprout J. M. L. Pentkowski N. S.. (2020). Preclinical support for the therapeutic potential of zolmitriptan as a treatment for cocaine use disorders. Transl. Psychiatry 10:266. 10.1038/s41398-020-00956-6 32747623 PMC7398918

[B16] Hecht G. S. Spear N. E. Spear L. P. (1999). Changes in progressive ratio responding for intravenous cocaine throughout the reproductive process in female rats. Dev. Psychobiol. 35, 136–145. 10.1002/(SICI)1098-2302(199909)35:2<136::AID-DEV6>3.0.CO;2-K 10461127

[B225] Heesch C. M. Negus B. H. Bost J. E. Keffer J. H. Snyder II R. W. Eichhorn E. (1996). Effects of cocaine on anterior pituitary and gonadal hormones. J. Pharmacol. Exp. Ther. 278, 1195–1200. 8819502

[B17] Hodos W. (1961). Progressive ratio as a measure of reward strength. Science 134, 943–944. 10.1126/science.134.3483.943 13714876

[B18] Hu M. Crombag H. S. Robinson T. E. Becker J. B. (2004). Biological basis of sex differences in the propensity to self-administer cocaine. Neuropsychopharmacology 29, 81–85. 10.1038/sj.npp.1300301 12955098

[B19] Jackson L. R. Robinson T. E. Becker J. B. (2006). Sex differences and hormonal influences on acquisition of cocaine self-administration in rats. Neuropsychopharmacology 31, 129–138. 10.1038/sj.npp.1300778 15920500

[B249] Killeen P. R. Reilly M. P. (2001). No thanks, I’m good. Any more and I’ll be sick: comment on Lynch and Carroll (2001). Exp. Clin. Psychopharmacol. 9, 144–144. 10.1037/1064-1297.9.2.144 11518087

[B20] King T. S. Schenken R. S. Kang I. S. Javors M. A. Riehl R. M. (1990). Cocaine disrupts estrous cyclicity and alters the reproductive neuroendocrine axis in the rat. Neuroendocrinology 51, 15–22. 10.1159/000125310 2106083

[B21] Kippin T. E. Fuchs R. A. Mehta R. H. Case J. M. Parker M. P. Bimonte-Nelson H. A.. (2005). Potentiation of cocaine-primed reinstatement of drug seeking in female rats during estrus. Psychopharmacology (Berl) 182, 245–252. 10.1007/s00213-005-0071-y 16001116

[B22] Lacy R. T. Strickland J. C. Feinstein M. A. Robinson A. M. Smith M. A. (2016). The effects of sex, estrous cycle and social contact on cocaine and heroin self-administration in rats. Psychopharmacology (Berl) 233, 3201–3210. 10.1007/s00213-016-4368-9 27370020 PMC5259804

[B24] Lee M. D. Simansky K. J. (1997). CP-94,253: a selective serotonin 1B (5-HT_1B_) agonist that promotes satiety. Psychopharmacology (Berl) 131, 264–270. 10.1007/s002130050292 9203237

[B25] Lynch W. J. (2006). Sex differences in vulnerability to drug self-administration. Exp. Clin. Psychopharmacol. 14, 34–41. 10.1037/1064-1297.14.1.34 16503703

[B26] Lynch W. J. (2008). Acquisition and maintenance of cocaine self-administration in adolescent rats: effects of sex and gonadal hormones. Psychopharmacology (Berl) 197, 237–246. 10.1007/s00213-007-1028-0 18066534

[B32] Lynch W. J. Arizzi M. N. Carroll M. E. (2000). Effects of sex and the estrous cycle on regulation of intravenously self-administered cocaine in rats. Psychopharmacology (Berl) 152, 132–139. 10.1007/s002130000488 11057516

[B27] Lynch W. J. Carroll M. E. (1999). Sex differences in the acquisition of intravenously self-administered cocaine and heroin in rats. Psychopharmacology (Berl) 144, 77–82. 10.1007/s002130050979 10379627

[B28] Lynch W. J. Carroll M. E. (2000). Reinstatement of cocaine self-administration in rats: sex differences. Psychopharmacology (Berl) 148, 196–200. 10.1007/s002130050042 10663435

[B29] Lynch W. J. Carroll M. E. (2001). Regulation of drug intake. Exp. Clin. Psychopharmacol. 9, 131–143. 10.1037//1064-1297.9.2.131 11518086

[B30] Lynch W. J. Taylor J. R. (2004). Sex differences in the behavioral effects of 24-h/day access to cocaine under a discrete trial procedure. Neuropsychopharmacology 29, 943–951. 10.1038/sj.npp.1300389 14872204

[B31] Lynch W. J. Taylor J. R. (2005). Decreased motivation following cocaine self-administration under extended access conditions: effects of sex and ovarian hormones. Neuropsychopharmacology 30, 927–935. 10.1038/sj.npp.1300656 15647749

[B243] Mello N. K. Mendelson J. H. Drieze J. Kelly M. (1990). Acute effects of cocaine on prolactin and gonadotropins in female rhesus monkey during the follicular phase of the menstrual cycle. J. Pharmacol. Exp. Ther. 254, 815–823. 2118570

[B33] Mello N. K. Negus S. S. (1996). Preclinical evaluation of pharmacotherapies for treatment of cocaine and opioid abuse using drug self-administration procedures. Neuropsychopharmacology 14, 375–424. 10.1016/0893-133X(95)00274-H 8726752

[B247] Mello N. K. Sarnyai Z. Mendelson J. H. Drieze J. M. Kelly M. (1993). Acute effects of cocaine on anterior pituitary hormones in male and female rhesus monkeys. J. Pharmacol. Exp. Ther. 266, 804–811. 8355210

[B242] Mendelson J. .H Sholar J. W. Seigel A. J. Mello N. K. (2001). Effect of cocaine on luteinizing hormone in women during the follicular and luteal phases of the menstrual cycle and in men. J. Pharmacol. Exp. Ther. 296, 972–979. 11181931

[B34] Miszkiel J. Filip M. Przegaliñski E. (2011). Role of serotonin 5-HT_1B_ receptors in psychostimulant addiction. Pharmacol. Rep. 63, 1310–1315. 10.1016/s1734-1140(11)70695-8 22358079

[B35] Parsons L. H. Weiss F. Koob G. F. (1998). Serotonin_1B_ receptor stimulation enhances cocaine reinforcement. J. Neurosci. 18, 10078–10089. 10.1523/JNEUROSCI.18-23-10078.1998 9822762 PMC6793270

[B36] Peartree N. A. Hatch K. N. Goenaga J. G. Dado N. R. Molla H. Dufwenberg M. A.. (2017). Social context has differential effects on acquisition of nicotine self-administration in male and female rats. Psychopharmacology (Berl) 234, 1815–1828. 10.1007/s00213-017-4590-0 28361264 PMC5451305

[B37] Pentkowski N. S. Acosta J. I. Browning J. R. Hamilton E. C. Neisewander J. L. (2009). Stimulation of 5-HT_1B_ receptors enhances cocaine reinforcement yet reduces cocaine-seeking behavior. Addict. Biol. 14, 419–430. 10.1111/j.1369-1600.2009.00162.x 19650818 PMC2846432

[B38] Pentkowski N. S. Cheung T. H. C. Toy W. A. Adams M. D. Neumaier J. F. Neisewander J. L. (2012). Protracted withdrawal from cocaine self-administration flips the switch on 5-HT_1B_ receptor modulation of cocaine abuse-related behaviors. Biol. Psychiatry 72, 396–404. 10.1016/j.biopsych.2012.03.024 22541946 PMC4071622

[B39] Pentkowski N. S. Harder B. G. Brunwasser S. J. Bastle R. M. Peartree N. A. Yanamandra K.. (2014). Pharmacological evidence for an abstinence-induced switch in 5-HT_1B_ receptor modulation of cocaine self-administration and cocaine-seeking behavior terms of use. ACS Chem. Neurosci. 5, 168–176. 10.1021/cn400155t 24369697 PMC3986226

[B240] Piazza P. V. Deroche-Gamonent V. Rouge-Pont F. Le Moal M. (2000). Vertical shifts in self-administration dose-response functions predict a drug-vulnerable phenotype predisposed to addiction. J. Neurosci. 20, 4226–4232. 10.1523/JNEUROSCI.20-11-04226.2000 10818158 PMC6772616

[B40] Pockros L. A. Pentkowski N. S. Swinford S. E. Neisewander J. L. (2011). Blockade of 5-HT_2A_ receptors in the medial prefrontal cortex attenuates reinstatement of cue-elicited cocaine-seeking behavior in rats. Psychopharmacology (Berl) 213, 307–320. 10.1007/s00213-010-2071-9 21079923 PMC3072217

[B41] Pomara C. Cassano T. D’Errico S. Bello S. Romano A. D. Riezzo I.. (2012). Data available on the extent of cocaine use and dependence: biochemistry, pharmacologic effects and global burden of disease of cocaine abusers. Curr. Med. Chem. 19, 5647–5657. 10.2174/092986712803988811 22856655

[B42] Richardson N. R. Roberts D. C. (1996). Progressive ratio schedules in drug self-administration studies in rats: a method to evaluate reinforcing efficacy. J. Neurosci. Methods 66, 1–11. 10.1016/0165-0270(95)00153-0 8794935

[B43] Roberts D. C. Bennett S. A. Vickers G. J. (1989). The estrous cycle affects cocaine self-administration on a progressive ratio schedule in rats. Psychopharmacology (Berl) 98, 408–411. 10.1007/BF00451696 2501818

[B48] Scott S. N. Garcia R. Powell G. L. Doyle S. M. Ruscitti R. Le T.. (2021). 5-HT_1B_ receptor agonist attenuates cocaine self-administration after protracted abstinence and relapse in rats. J. Psychopharmacol. 35, 1216–1225. 10.1177/02698811211019279 34049460

